# Diets and selected lifestyle practices of self-defined adult vegetarians from a population-based sample suggest they are more 'health conscious'

**DOI:** 10.1186/1479-5868-2-4

**Published:** 2005-04-13

**Authors:** Jennifer L Bedford, Susan I Barr

**Affiliations:** 1 Human Nutrition, University of British Columbia, 2205 East Mall, Vancouver, BC, Canada

**Keywords:** Diet, vegetarian, Health behavior, Food habits, Health attitudes and behaviors.

## Abstract

**Background:**

Few population-based studies of vegetarians have been published. Thus we compared self-reported vegetarians to non-vegetarians in a representative sample of British Columbia (BC) adults, weighted to reflect the BC population.

**Methods:**

Questionnaires, 24-hr recalls and anthropometric measures were completed during in-person interviews with 1817 community-dwelling residents, 19–84 years, recruited using a population-based health registry. Vegetarian status was self-defined. ANOVA with age as a covariate was used to analyze continuous variables, and chi-square was used for categorical variables. Supplement intakes were compared using the Mann-Whitney test.

**Results:**

Approximately 6% (n = 106) stated that they were vegetarian, and most did not adhere rigidly to a flesh-free diet. Vegetarians were more likely female (71% vs. 49%), single, of low-income status, and tended to be younger. Female vegetarians had lower BMI than non-vegetarians (23.1 ± 0.7 (mean ± SE) vs. 25.7 ± 0.2 kg/m^2^), and also had lower waist circumference (75.0 ± 1.5 vs. 79.8 ± 0.5 cm). Male vegetarians and non-vegetarians had similar BMI (25.9 ± 0.8 vs. 26.7 ± 0.2 kg/m^2^) and waist circumference (92.5 ± 2.3 vs. 91.7 ± 0.4 cm). Female vegetarians were more physically active (69% vs. 42% active ≥4/wk) while male vegetarians were more likely to use nutritive supplements (71% vs. 51%). Energy intakes were similar, but vegetarians reported higher % energy as carbohydrate (56% vs. 50%), and lower % protein (men only; 13% vs. 17%) or % fat (women only; 27% vs. 33%). Vegetarians had higher fiber, magnesium and potassium intakes. For several other nutrients, differences by vegetarian status differed by gender. The prevalence of inadequate magnesium intake (% below Estimated Average Requirement) was lower in vegetarians than non-vegetarians (15% vs. 34%). Female vegetarians also had a lower prevalence of inadequate thiamin, folate, vitamin B_6 _and C intakes. Vegetarians were more likely than non-vegetarians to consider various health conditions and food/nutrition concerns when choosing foods.

**Conclusion:**

In this population-based study, evidence was obtained to indicate that vegetarians appear more 'health conscious' than non-vegetarians, although specific differences were not always consistent by gender. Additional population-based studies are required to determine if the observed gender differences exist in other populations.

## Background

Interest in the dietary habits of vegetarians emerges from research suggesting that vegetarians have a lower prevalence of various chronic diseases that currently plague the developed world [1,2]. It has been hypothesized that these findings are due to vegetarians' dietary habits, which more closely follow recommendations for healthy eating [3], and also to their lower BMI [4]. Recently, however, it has been found that mortality rates did not differ among vegetarians and similar 'health conscious' omnivores [5,6] despite vegetarians' lower age-adjusted BMI [5-7]. This suggests that other lifestyle behaviors commonly observed in health conscious individuals may be responsible for the observed beneficial health effects. Yet, it is important to note that the majority of reports of vegetarians' dietary intakes and lifestyle behaviors are from convenience samples.

Few population-based studies have examined vegetarians' dietary intake and habits. Kennedy and coworkers used population representative data from the Continuing Survey of Food Intakes by Individuals (CSFII) to gain insights into the dietary patterns of vegetarians [8]. Specifically, they compared the intakes of those who consumed meat on data collection days to those who did not. However, many of those who did not consume meat were likely not 'vegetarian' as the proportion of those grouped as vegetarian by this method (15%) was much higher than those who self-identified as vegetarian in the same sample (2.5%) [9].

CSFII data were also analyzed to compare self-identified vegetarians to non-vegetarians [9]. Adult vegetarians were found to have lower BMI as well as lower intakes of total fat, saturated fat and cholesterol and higher intakes of fiber and fruits [9]. However, differences by gender or age were not examined, and if age or gender differences in the prevalence of vegetarian status exist, as has been observed in other population representative studies of vegetarians [10], these could confound the results. For example, if vegetarians were more likely than non-vegetarians to be younger and female this could impact group BMIs and intakes of nutrients that differ by gender or age. In addition, differences in lifestyle behaviors were not assessed. This could be important to consider as it has been suggested that other lifestyle behaviors may be the determinant of differences observed in health conditions by vegetarian status [5,6].

Thus, the aim of the present analysis was to describe and compare the demographics, lifestyle behaviors, dietary intake, nutritive supplement use, and food and nutrition concerns of male and female self-defined vegetarians and non-vegetarians from a population-based representative sample of adults from the province of British Columbia, Canada.

## Methods

The data used for this analysis were collected as part of the British Columbia Nutrition Survey (BCNS). Details of the methodology used for the BCNS including sampling strategies, survey instruments, and data entry and analysis are described in detail elsewhere [11], and are summarized below.

### Participants

Adults aged 19 to 84 years living in BC were recruited for the BCNS using the BC Health Registry – a central repository of individuals who receive health services in BC. Exclusion criteria included those living in care or correctional facilities, military bases, or Indian Reserves, as well as pregnant and lactating women. Less than 3% of the population was excluded on these grounds. The sample was stratified by age, sex and geographical region. The study protocol was approved by the University of British Columbia's Behavioral Research Ethics Board, and written informed consent was obtained from all participants.

### Measures

The BCNS included a 24-hour recall; questions on food habits and choices, physical activity and demographics; and anthropometric measurements.

#### 24-Hour Recall

To obtain information on dietary intake each individual completed a 24-hour recall conducted by personal interview using the open-ended, multiple-pass technique for which each participant recalled all foods and beverages consumed on the previous day (midnight to midnight). Food models and household measures were used to estimate portion sizes. In one third of the sample, a second 24-hour recall was conducted on a different weekday at least one week following the first recall. Supplement data were also obtained during the 24-hour recall. Participants were first asked whether they took any nutritional supplements yesterday followed by a question about whether they took any supplements within the past month that differed from the ones taken yesterday. The frequency (daily, weekly or monthly) and the number or amount of each supplement were also recorded. When possible, brand names and the drug identification number (DIN) were recorded.

#### Food and Nutrition Habits

Participants were read a list of health-related reasons for choosing and avoiding foods to determine nutrition and food concerns. Vegetarian status was assessed by asking participants if they considered themselves to be a vegetarian. Those who answered 'yes' were also asked if they ever ate animal products including dairy, eggs, fish/seafood, poultry and red meat.

#### Physical Activity

Questions from previously validated instruments were asked to obtain information on physical activity, including the frequency of mild, moderate and strenuous activity; and motivational readiness for exercise [12,13].

#### Demographic Questionnaire

Questions regarding age, gender, marital status, education and income were asked to characterize the sample.

#### Anthropometrics

Weight, without shoes, hats or any heavy clothing or items, was measured using a weekly calibrated electronic scale. Height was measured using a setsquare and measuring tape, and girths were assessed using a measuring tape. Weight and height were measured and recorded once; waist circumferences was measured and recorded at least twice. BMI was calculated from weight and height (kg/m^2^).

### Procedure

Eligible residents who chose not to participate were asked to complete a non-response questionnaire to determine if non-responders differed from those who agreed to participate. Those who agreed to participate were interviewed in person by trained interviewers. Interviews lasted approximately 90 minutes and most took place in participants' homes.

### Analysis

#### Response rate

In a large population survey such as the BCNS, the response rate depends on whether individuals that could not be contacted (unresolved cases) were eligible to participate in the survey. Lower and upper bounds for the response rate can be calculated based on the assumption that all unresolved cases are eligible (lower bound), or that all unresolved cases are ineligible (upper bound).

#### Nutrient intake data

All data were sent to the Bureau of Nutritional Sciences at Health Canada and were entered into the Nutrition Survey System, a software program that uses the Canadian Nutrient File and a recipe database adapted from the United States Department of Agriculture CSFII. The Canadian Nutrient File was updated to reflect fortification of grain products with folic acid that began in Canada in 1998. Data on nutritional supplements were entered using the DIN or by name and/or nutrient content. The method of estimating the distribution of usual intakes from food sources alone and from food sources and supplements used the data from duplicate 24-hour recalls to remove within-person variability from population distributions of nutrient intakes, yielding an adjusted distribution of usual intakes for age-sex groups [11,14]. Then the monthly supplement data were expressed per day and added to the adjusted usual intake distribution. The proportion of this distribution that fell below the Estimated Average Requirement (EAR) was used to estimate the prevalence of inadequate nutrient intakes in an age-sex group, for nutrients with an EAR and a symmetrical requirement distribution [14]. This analysis could not be conducted for vitamin A as the EAR is expressed in new Retinol Activity Equivalents, whereas intake data were in Retinol Equivalents. Food intake data were also expressed as number of servings from the food groups included in Canada's Food Guide to Healthy Eating (CFGHE).

### Statistical analysis

All data were weighted to reflect the BC population based on gender, age, and geographical region, and were analyzed using the Statistical Package for Social Sciences (SPSS; v11.0, Chicago, Ill., 2002). One-way ANOVA with age as a covariate was used to analyze parametric data (demographics, nutrient intakes) and Chi-square analysis was used to analyze categorical data (lifestyle behaviors, supplement usage, nutrition concerns) between vegetarians and non-vegetarians. The Mann-Whitney test was used to compare differences by vegetarian status in supplemental nutrient intake as the data were not normally distributed. Non-parametric tests were also applied to nutrient intake data that were not normally distributed. However, findings did not differ from those of parametric analysis and thus ANOVA was applied to all nutrients for consistency, and because ANOVA allowed consideration of effects of covariates. The data were also examined to assess whether demographic differences between vegetarians and non-vegetarians (other than age and sex) may have influenced the results. The significance level was set at p = 0.05 for all statistical measures.

## Results

Response rate lower and upper bounds were 42% and 52%, respectively [11]. Approximately 66% of those who declined to participate completed the non-respondent survey. Using this information, it was found that BCNS participants were less likely to smoke (17% vs. 23%) and more likely to use vitamin/mineral supplements (66% vs. 60%) and hold university degrees than non-participants (14% vs. 9%). However, in comparison to the general BC population, BCNS participants had a similar prevalence of smoking, although study participants were more educated (21% vs. 13% completed university) and more men were married (64% vs. 55%). Because of the difference in educational attainment between survey participants and the population, the effect of this variable on nutrient intakes was examined. Educational attainment was associated only with the intakes of vitamin C and vitamin B_12_: it appeared that those with more education had higher intakes of vitamin C and lower intakes of vitamin B_12 _[11].

Of the 1817 participants, 5.8% (n = 106) identified themselves as vegetarian. Those who identified themselves as vegetarian were asked if they 'ever' ate various animal products. It appeared that the majority of self-identified vegetarians did not adhere rigidly to a vegetarian dietary pattern: In terms of tissue protein, 74.9% consumed fish and/or seafood at least occasionally, while 57.6% consumed poultry and 22.4% consumed red meat at least occasionally. Dairy products were used at least occasionally by 97.3% and eggs by 92.3%.

### Demographics

The demographic characteristics of vegetarian and non-vegetarian participants are presented in Table 1. Vegetarians tended to be younger than non-vegetarians (p = 0.057), and the age group distribution differed significantly, with more vegetarians falling in the 19 to 30 year range. Groups differed in sex distribution with women representing over 70% of vegetarians and half of non-vegetarians. Although the majority of both groups was married, there was a significant difference in marital status distribution, with vegetarians more likely to be single. In addition, vegetarians were significantly more likely to be of low income status although there were no differences in education level. Accordingly, data were examined to assess the effects of marital status and income status.

**Table 1:** Participant demographics by vegetarian status

	*Non-vegetarian (n = 1711)*	*Vegetarian (n = 106)*	*Test Statistic*^1^	*P value*
Age (years, mean ± SE)	44.8 ± 0.4	41.5 ± 1.8	F = 3.63	0.057

Age Group			χ^2 ^= 9.80	0.020
19 – 30 years	24.8%	38.1%		
31 – 50 years	40.9%	31.4%		
50 – 70 years	24.3%	20.0%		
71+ years	10.1%	10.5%		

Sex (% female)	49.2%	70.8%	χ^2 ^= 18.58	<0.001

Marital status			χ^2 ^= 17.55	0.001
Single	22.5%	35.2%		
Married	62.7%	50.5%		
Widowed	5.3%	10.5%		
Divorced/separated	9.5%	3.8%		

Education level			χ^2 ^= 0.45	0.800
Secondary or less	33.3%	32.1%		
Technical or some university	47.6%	46.2%		
University graduate	19.1%	21.7%		

Low income	22.8%	37.2%	χ^2 ^= 9.28	0.002

Weight (kg, mean ± SE)				
Men	83.1 ± 0.6	82.2 ± 3.0	F = 0.10	0.754
Women	67.9 ± 0.6	62.5 ± 1.8	F = 7.92	0.005

BMI (kg/m^2^, mean ± SE)				
Men	26.7 ± 0.2	25.9 ± 0.8	F = 0.81	0.346
Women	25.7 ± 0.2	23.1 ± 0.7	F = 13.66	<0.001

BMI category – men			χ^2 ^= 0.92	0.821
Underweight (< 18)	0.2%	0.0%		
Normal weight (18-<25)	48.8%	51.6%		
Overweight (≥ 25-<30)	31.6%	35.5%		
Obese (≥ 30)	19.4%	12.9%		

BMI category – women			χ^2 ^= 16.34	0.001
Underweight (< 18)	1.1%	0.0%		
Normal weight (18-<25)	59.6%	83.1%		
Overweight (≥ 25-<30)	21.2%	12.7%		
Obese (≥ 30)	18.1%	4.2%		

Waist Circ. (cm, mean ± SE)				
Men	91.7 ± 0.4	92.5 ± 2.3	F = 0.11	0.740
Women	79.8 ± 0.5	75.0 ± 1.5	F = 9.66	0.002

Health Condition – Men (% yes)				
Diabetes	5.5	3.2	χ^2 ^= 0.31	0.580
Heart disease	6.1	19.4	χ^2 ^= 8.59	0.003
Stroke	0.8	3.2	χ^2 ^= 1.99	0.158
High blood pressure	11.6	6.5	χ^2 ^= 0.79	0.375
High cholesterol	14.7	9.7	χ^2 ^= 0.61	0.434
Cancer	4.5	6.5	χ^2 ^= 0.27	0.606
Osteoporosis	0.7	0.0	χ^2 ^= 0.22	0.643
None of the above	70.9	63.3	χ^2 ^= 0.81	0.370

Health Condition – Women (% yes)				
Diabetes	4.9	1.3	χ^2 ^= 1.97	0.160
Heart disease	3.7	4.0	χ^2 ^= 0.02	0.889
Stroke	1.9	0.0	χ^2 ^= 1.45	0.228
High blood pressure	15.3	6.7	χ^2 ^= 4.15	0.042
High cholesterol	11.3	6.8	χ^2 ^= 1.44	0.230
Cancer	8.3	1.3	χ^2 ^= 4.70	0.030
Osteoporosis	6.1	8.0	χ^2 ^= 0.44	0.506
None of the above	67.7	78.4	χ^2 ^= 3.62	0.057

^1. ^Statistical analysis consisted of ANOVA (vegetarian versus non-vegetarian, with age as a covariate) for continuous variables. Chi-square analysis was used for categorical variables.

Female vegetarians had a significantly lower mean age-adjusted body weight and mean BMI than non-vegetarians, as well as a lower waist circumference. Low income status and marital status did not affect these variables. In addition, vegetarian women were significantly less likely to be classified as overweight or obese (17% vs. 40%). Conversely, for males, weight, BMI, and BMI category distribution were very similar between vegetarians and non-vegetarians, with approximately 50% of both groups classified as overweight or obese. There were no significant differences in age-adjusted waist circumference between vegetarian and non-vegetarian men.

The prevalence of certain health conditions differed by vegetarian status. Male vegetarians had a higher prevalence of heart disease while female non-vegetarians were more likely to report cancer and hypertension.

### Lifestyle behaviors

Female vegetarians were more likely than non-vegetarians to report moderate to strenuous physical activity four or more times weekly (69% vs. 42%, χ^2 ^= 21.69, p < 0.001), and more women vegetarians than non-vegetarians were in the 'action' or 'maintenance' stages of motivational readiness for exercise (76% vs. 53%, χ^2 ^= 21.67, p < 0.001). Although single women in the sample as a whole were more active than women who were married, widowed, divorced or separated, when age was considered marital status did not affect physical activity level. Low income status was not associated with physical activity in women. In contrast, male vegetarians and non-vegetarians did not differ in the amount of weekly exercise: the majority of both groups participated in moderate to strenuous physical activity less than four times a week (55% vs. 52% respectively, χ^2 ^= 0.18, p = 0.913). Men were also similar in terms of the distribution of the stage of motivational readiness for exercise (χ^2 ^= 1.78, p < 0.776). On the other hand, smoking status differed between vegetarians and non-vegetarians for both men (3% vs. 18%, χ^2 ^= 4.15, p < 0.05) and women (0% vs. 18%, χ^2 ^= 15.85, p < 0.001).

### Supplement use and intakes

The majority of all groups reported nutritive supplement use. Among men, significantly more vegetarians than non-vegetarians reported using supplements (71% vs. 51%, χ^2 ^= 4.76, p = 0.029). However, for women, the difference was not significant with 76% of vegetarians and 68% of non-vegetarians reporting supplement use (χ^2 ^= 1.88, p = 0.170). On the other hand, female vegetarians who used supplements reported using a higher number of supplements than non-vegetarians (3.5 ± 0.4 (mean ± SE) vs. 2.3 ± 0.1, F = 15.33, p < 0.001), while the numbers used by males were similar (1.3 ± 0.4 vs. 1.5 ± 0.1, F = 0.40, p = 0.53).

The proportion of individuals using supplements of many nutrients differed significantly by vegetarian status (data not shown). More vegetarians than non-vegetarians of both sexes used a supplement containing the B vitamins. However, for other nutrients, the results were split along gender lines: more female vegetarians used supplements of all other vitamins/minerals except for vitamin E and calcium; whereas among males, there were no additional significant differences by vegetarian status.

Differences in supplemental nutrient intake were also evident between vegetarians and non-vegetarians who used supplements (data not shown). Among women, vegetarians had significantly higher median supplemental intakes of calcium, iron, magnesium, potassium, niacin, folic acid and vitamins A, D and B_12_. Among men, vegetarians had a significantly higher median supplemental intake of vitamin C.

### Energy and nutrient intakes

Age-adjusted energy and nutrient intakes from food are presented by vegetarian status and gender (see Additional File 1). Income status did not affect nutrient intakes [11]. There were no significant differences in energy intake between vegetarians and non-vegetarians, but energy distribution differed significantly by vegetarian status for both sexes. Compared to non-vegetarians, both male and female vegetarians consumed significantly more energy as carbohydrate. Among men, vegetarians had a significantly lower proportion of energy from protein. Conversely, female vegetarians had a significantly lower percentage of energy from fat.

Male and female vegetarians had significantly higher intakes of fiber, magnesium and potassium. Female vegetarians had significantly higher intakes of carbohydrate (g), phosphorus, thiamin, pantothenic acid, vitamin B_6_, and folate and lower intakes of saturated fat and sodium. Conversely, male vegetarians had significantly higher intakes of vitamin C and calcium, and lower intakes of protein (g), niacin and cholesterol.

**Figure 1:**
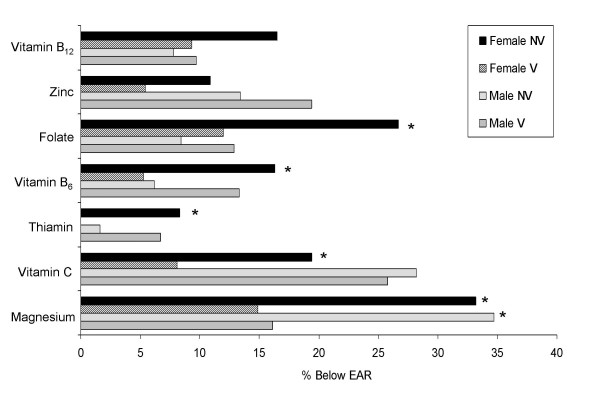
Prevalence of nutrient inadequacies by vegetarian status and gender for selected nutrients. The prevalence of nutrient inadequacy was estimated by determining the proportion of the usual intake distribution (from food plus supplements) that was below the Estimated Average Requirement (EAR). * Prevalence of inadequacy higher in non-vegetarians (p < 0.05). NV = non-vegetarians, V = vegetarians.

The prevalence of inadequate intakes of selected nutrients by vegetarian status and gender are shown in Figure 1. These data are based on combined intake from food plus supplements. No differences were observed in the prevalence of inadequate intakes of vitamin B_12 _or zinc, but significantly more non-vegetarians had intakes below the EAR for magnesium in both men and women. There were also significant differences for both genders in the prevalence of inadequacies for thiamin, although in both cases the prevalence of inadequacy was <10%: for men, more vegetarians were below the EAR, while for women, non-vegetarians were more likely to be below the EAR. Female non-vegetarians were also significantly more likely to be below the EAR for vitamin C, vitamin B_6 _and folate.

### Intake based on Canada's Food Guide to Healthy Eating

Analyses of dietary intake based on servings of CFGHE food groups by vegetarian status and gender, adjusted for age, are presented in Table 2. Vegetarians of both genders had a significantly higher number of servings of fruits and vegetables. Only female vegetarians had a significantly higher number of servings of grain products while only male vegetarians had a significantly higher number of servings of milk products and a significantly lower number of servings of meat and alternatives.

**Table 2:** Canada's Food Guide servings and percentage of participants meeting minimum recommendations by vegetarian status and gender.

*Food Group*	*Servings (Mean ± SE)*		*% Meeting Recommendation*	
	Non-vegetarian	Vegetarian	Non-vegetarian	Vegetarian

Grain Products^1^				
♂	7.7 ± 0.16	7.9 ± 0.81	70.5	67.7
♀	5.0 ± 0.09	5.9 ± 0.31**	45.5	57.3*

Fruit and Vegetables^1^				
♂	5.3 ± 0.13	7.3 ± 0.70**	45.5	64.5*
♀	4.5 ± 0.12	5.6 ± 0.40**	34.0	40.0

Milk and Milk Products^2^				
♂	1.7 ± 0.06	3.0 ± 0.29***	33.5	67.7***
♀	1.4 ± 0.04	1.5 ± 0.14	25.3	33.3

Meat and Alternatives^3^				
♂	4.5 ± 0.12	1.9 ± 0.6***	78.1	51.6***
♀	2.7 ± 0.07	2.6 ± 0.24	57.8	45.3*

* p < 0.05, ** p < 0.01, *** p < 0.001

^1 ^Minimum serving recommendation is 5 servings.

^2 ^Minimum serving recommendation is 2 servings.

^3 ^Minimum serving recommendation is 2 servings; servings calculated as 50 g equivalent

The proportions of participants meeting the minimum number of CFGHE servings by vegetarian status and gender are also displayed in Table 2. Vegetarians of both genders were less likely to meet the minimum recommendations for meat and alternatives. Among women, vegetarians were more likely to meet the minimum servings of grain products, while among men, vegetarians were more likely to meet recommendations for fruits and vegetables as well as milk products.

### Food and nutrition concerns

For both genders, vegetarians were significantly more likely to report 'maintaining/improving health' as a consideration when choosing/avoiding foods than non-vegetarians (men: 100% vs. 65%, p < 0.001; women: 93% vs. 77%, p = 0.001). Male vegetarians were more likely than non-vegetarians to also consider heart disease (77% vs. 38%, p < 0.001) and high blood pressure (45% vs. 25%, p = 0.013) when choosing/avoiding foods. Female vegetarians were more likely than non-vegetarians to also consider cancer (41% vs. 30%, p = 0.05), osteoporosis (61% vs. 38%, p < 0.001) and food allergies/intolerances (43% vs. 30%, p = 0.026), and were less likely than non-vegetarians to consider weight gain (46% vs. 61%, p = 0.013) when choosing/avoiding foods. Finally, more non-vegetarians than vegetarians reported that they did not consider any of the aforementioned factors (maintaining/improving health, heart disease, cancer, osteoporosis, high blood pressure, weight gain, food allergies/intolerances) when choosing/avoiding foods (men: 26% vs. 0%, p = 0.001; women: 12% vs. 1%, p = 0.004).

Vegetarians of both genders were more likely than non-vegetarians to report choosing foods because of the nutrients they contain (men: 73% vs. 53%, p = 0.025; women: 88% vs. 68%, p < 0.001), and to report avoiding foods because of their fat content (men: 77% vs. 59%, p = 0.041; women: 84% vs. 72%, p = 0.023). Male vegetarians were more likely than non-vegetarians to also consider the type of fat (63% vs. 36%, p = 0.003) and the amount of unsaturated fat (63% vs. 28%, p < 0.001) when choosing foods and to avoid foods because of their cholesterol (55% vs. 36%, p = 0.032) and saturated fat (58% vs. 38%, p = 0.021) content. Female vegetarians were more likely than non-vegetarians to also report avoiding foods because of their salt content (57% vs. 45%, p = 0.043). On the other hand, more non-vegetarians than vegetarians reported that, when choosing foods, they did not consider *any *of nutrient content, type of fat, amount of unsaturated fat, or fiber content (men: 34% vs. 16%, p = 0.035; women: 19% vs. 3%, p < 0.001). When avoiding foods, more female non-vegetarians reported that they did not consider *any *of the fat, salt, cholesterol, sugar or saturated fat content (16% vs. 5%, 0 = 0.014).

## Discussion

The purpose of this study was to examine and compare the dietary habits and lifestyle behaviors of self-defined vegetarians and non-vegetarians from a population-based representative sample of BC adults. Approximately 6% of the sample, weighted to reflect the BC population, reported being vegetarian. The findings of this study suggest that the dietary habits, lifestyle behaviors, and food-choice motivations of self-defined vegetarians differ from those of non-vegetarians, and that there may be variation between men and women which has not previously been examined in population-based studies.

Several aspects of our results warrant additional consideration, one of which is the small proportion of self-identified vegetarians who adhered rigidly to diets free from animal flesh. Occasional use of seafood, poultry, or meat by a majority of those who consider themselves to be vegetarian has also been reported in other studies [9,15]. If a strict definition of vegetarianism had been used, the prevalence in our study would be less than 1.5% rather than close to 6%. Despite basing our analysis on respondents' self-definition, we still observed a number of differences in nutrient intake and lifestyle behavior. At some level, this validates respondents' self-identification as vegetarian.

Evidence for a higher level of 'health consciousness' among vegetarians in our sample was provided by findings of increased use of nutrient supplements, higher intakes of several nutrients (fiber, magnesium, potassium), higher intakes of fruits and vegetables, a considerably lower prevalence of smoking, and among women, higher physical activity and a lower BMI. Many of these findings have been reported in other studies, although most reports from convenience samples have not found differences in smoking or exercise behavior by vegetarian status [7,16-21]. It is likely that convenience sampling resulted in recruitment of more 'health conscious' participants and therefore did not detect differences. Thus our findings provide population-level support for the concept that vegetarians have healthier lifestyle practices than the general population of non-vegetarians.

Vegetarians were also more likely to consider 'maintaining/improving health' when choosing/avoiding foods, to choose foods for the nutrients they contain and to avoid foods for their fat content. These findings provide additional evidence of health consciousness, and are consistent with research reporting that health concerns and benefits are a primary reason for adopting a vegetarian lifestyle [22,23], although we did not assess motivation for adopting a vegetarian diet. They are also consistent with a population-based study in the Netherlands that found vegetarians were more likely to report health considerations when purchasing food [10]. That study, however, did not report nutrient intakes.

A novel aspect of our analysis was that, in addition to assessing differences in nutrient intakes, we also compared the prevalence of inadequate nutrient intakes using the EAR cut-point method [14]. As assessed by the proportions with total usual nutrient intakes below the EAR, vegetarians were less likely to have an inadequate intake of magnesium, and female vegetarians were also less likely to have inadequate intakes of folate, vitamin C, thiamin and vitamin B_6_. Although there were no differences by vegetarian status in the proportions with zinc intakes below the EAR, this may not be an accurate reflection of zinc adequacy, as the requirement for dietary zinc may be as much as 50% greater for vegetarians [24]. Similarly, iron requirements of vegetarians are estimated to be 80% greater than those of non-vegetarians [24]. However, the adequacy of iron intakes was not assessed in our study because the iron requirement distribution is skewed, and therefore the EAR cut-point method cannot be used to estimate the prevalence of inadequacy [14]. Finally, although adequacy of vitamin B_12 _intakes is often identified as a concern for vegetarians, in our sample the prevalence of inadequate intakes was similar by vegetarian status. This is likely due to the fact that almost all vegetarians used dairy products and eggs, as well as to the high prevalence of B vitamin supplementation among vegetarians.

Although our vegetarian sample was small, our results provide suggestive evidence of gender differences. For example, vegetarian women had a lower age-adjusted BMI and waist circumference, and a lower prevalence of overweight/obesity, while no differences were seen between vegetarian and non-vegetarian men. This may have been due to the higher frequency of physical activity reported by vegetarian women (but not men), as energy intake did not differ by vegetarian status for either sex. Reports from convenience samples often suggest that vegetarians have lower BMI and/or a lower rate of obesity [2,7,22,25-27]. Conversely, other convenience samples, in which energy intakes and physical activity were similar between vegetarians and non-vegetarians, did not detect differences in BMI between groups [16-19,28,29]. In the population-based CSFII, self-identified vegetarians had lower energy intakes and age-adjusted BMI [9]. However, a major limitation of that report was that analyses were not conducted by gender. Accordingly, if vegetarians were more likely to be female, as observed in our sample and another population-based sample [10], vegetarians' mean energy intake and BMI would appear to be lower because of women's lower mean energy intakes and BMI.

The distribution of macronutrient intakes also provided suggestive evidence of gender differences. Carbohydrate as a percentage of energy was higher among vegetarians, as was also found in the CSFII vegetarian analysis [9] and the majority of convenience sample studies [18,22,26,27,30,31]. Other studies have also reported lower percentage energy from fat [8,9,22,27,32] and protein [8,18,22,27,28,30-32]. In our sample, only male vegetarians had a lower proportion of energy from protein and only female vegetarians consumed less energy from fat.

We also observed gender differences in motivations for choosing/avoiding foods. Only male vegetarians were more likely to report considering heart disease and high blood pressure when choosing/avoiding foods and to report avoiding foods because of their cholesterol or saturated fat content. This is consistent with the higher prevalence of heart disease among the male vegetarians in our sample, who we speculate may have chosen to follow a vegetarian diet as a *result *of heart disease. Because we did not assess motivation for adopting a vegetarian diet, this cannot be ascertained, and in any case, the study's cross-sectional design precludes causal inferences. Female vegetarians, on the other hand, were not more concerned about heart disease, but were more likely to consider cancer, osteoporosis and food allergies/intolerances when choosing/avoiding foods and to avoid foods because of their salt content. They were also less likely to consider weight gain when choosing/avoiding foods. It has been suggested that some young women may adopt a vegetarian lifestyle in an effort to lose weight [33,34]; however, this does not appear to be true for our population-based sample.

While our findings suggest that variation by gender may exist in vegetarians' dietary habits and lifestyle behaviors, the study limitations should be acknowledged. First, although the sample was considered representative of the province of British Columbia, it was not nationally representative, which means that inferences cannot be made about the Canadian population. Also, the response rate, although typical of other studies of this kind, was not optimal. Second, the absolute number of self-identified vegetarians was small and therefore caution must be used when interpreting the apparent gender differences. We had limited power to detect gender-by-vegetarian status interactions. Finally, data on dietary intake and lifestyle behaviors were based on self-reports, and it is known that dietary intakes are underreported [35]. This would be problematic if differences existed in the extent of underreporting by vegetarian status. However, based on similar reported energy intakes of the two groups, it appears unlikely that differential underreporting occurred.

We do not believe that our observations of higher 'health consciousness' among vegetarians were confounded by other differences between vegetarian and non-vegetarian groups. First, although the prevalence of vegetarianism was higher among women than men, we conducted analyses separately by gender. Second, vegetarians tended to be younger than non-vegetarians, so age was included as a covariate in nutrient intake and anthropometric analyses. Third, although vegetarians were more likely to be single and to report low-income status, consideration of these differences did not affect our observations.

## Conclusion

Taken together, these population-based findings add further support to the concept that adult vegetarians are more health-conscious than non-vegetarians, and that this difference extends to food choice and nutrition concerns. Additional population-based studies comparing dietary habits and lifestyle behaviors by vegetarian status and gender are needed to determine if gender differences observed in our representative sample exist in other populations in the developed world.

## Competing interests

The author(s) declare that they have no competing interests.

## Authors' contributions

JB performed the statistical analyses and drafted the manuscript. SB conceived of the study and participated in its design, and helped to draft the manuscript. Both authors read and approved the final manuscript.

## Supplementary Material

Additional File 1 Dietary intake (age-adjusted, mean ± SE) by vegetarian status and gender A Click here for file
