# Supplementary material for: Diets and selected lifestyle practices of self-defined adult vegetarians from a population-based sample suggest they are more 'health conscious'
Source: Int J Behav Nutr Phys Act. 2005 Apr 13;2:4. doi: 10.1186/1479-5868-2-4 (PMC1090609; doi:10.1186/1479-5868-2-4)
Supplement: Additional File 1 — Dietary intake (age-adjusted, mean ± SE) by vegetarian status and gender A [file 1479-5868-2-4-S1.doc]

Additional File 1. Dietary intake (age-adjusted, mean ± SE) by vegetarian status and gender

| Nutrient | Men | | | |  | Women | | | |
| --- | --- | --- | --- | --- | --- | --- | --- | --- | --- |
|  | Non-vegetarian | Vegetarian | Rec.^1^ | P |  | Non-vegetarian | Vegetarian | Rec.^1^ | P |
| Energy (MJ) | 10.9 ± 0.1 | 10.9 ± 0.1 | n/a | 0.968 |  | 7.5 ± 0.1 | 7.7 ± 0.3 | n/a | 0.416 |
| Carbohydrate (g) | 325 ± 4.6 | 348 ± 24.2 | 130^2^ | 0.349 |  | 228 ± 3.0 | 257 ± 10.0 | 130^2^ | 0.005 |
| (%energy) | 50.2 ± 0.4 | 56.1 ± 2.1 | 45-65%^3^ | 0.005 |  | 51.4 ± 0.4 | 56.9 ± 1.3 | 45-65%^3^ | <0.001 |
| Protein (g) | 107 ± 1.6 | 86 ± 8.4 | 56^1^ | 0.020 |  | 70 ± 1.0 | 71 ± 3.3 | 46 | 0.710 |
| (%energy) | 16.5 ± 0.2 | 13.0 ± 0.9 | 10-35%^3^ | <0.001 |  | 16.0 ± 0.5 | 15.3 ± 0.6 | 10-35%^3^ | 0.232 |
| Lipid (g) | 93.3 ± 2.1 | 92.9 ± 8.1 | n/a | 0.965 |  | 65.8 ± 1.2 | 59.0 ± 6.0 | n/a | 0.090 |
| (%energy) | 31.9 ± 0.3 | 30.6 ± 1.6 | 20-35%^3^ | 0.450 |  | 32.5 ± 0.3 | 27.7 ± 1.1 | 20-35%^3^ | <0.001 |
| Saturates (g) | 30.3 ± 0.6 | 29.6 ± 3.0 | n/a | 0.805 |  | 22.6 ± 0.5 | 15.2 ± 1.7 | n/a | <0.001 |
| Monunsaturates (g) | 37.7 ± 0.7 | 39.3 ± 3.6 | n/a | 0.662 |  | 26.1 ± 0.5 | 24.0 ± 1.7 | n/a | 0.214 |
| Polyunsaturates (g) | 16.6 ± 0.3 | 17.0 ± 1.8 | n/a | 0.824 |  | 10.9 ± 0.2 | 12.4 ± 0.8 | n/a | 0.063 |
| Cholesterol (mg) | 355 ± 9.5 | 236 ± 29.4 | n/a | 0.018 |  | 236 ± 6.7 | 209 ± 22.3 | n/a | 0.242 |
| Linoleate (g) | 13.6 ± 0.3 | 13.2 ± 1.5 | 14-17^4^ | 0.809 |  | 8.9 ± 0.2 | 9.8 ± 0.7 | 11-12^4^ | 0.190 |
| Linolenate (g) | 2.4 ± 0.08 | 2.8 ± 0.4 | 1.6^4^ | 0.325 |  | 1.8 ± 0.07 | 1.7 ± 0.24 | 1.1^4^ | 0.149 |
| Fiber (g) | 19.9 ± 0.4 | 29.4 ± 2.1 | 30-38^4^ | <0.001 |  | 15.5 ± 0.3 | 20.9 ± 1.1 | 21-25^4^ | <0.001 |
| Calcium (mg) | 980 ± 19.4 | 1559 ± 101 | 1000-1200^4^ | <0.001 |  | 777 ± 15 | 830 ± 50 | 1000-1200^4^ | 0.310 |
| Iron (mg) | 18.1 ± 0.3 | 19.1 ± 1.4 | 8^2^ | 0.497 |  | 12.8 ± 0.2 | 14.1 ± 0.6 | 8-18 | 0.056 |
| Magnesium (mg) | 393 ± 5.6 | 492 ± 29.3 | 400-420^2^ | 0.001 |  | 296 ± 4.4 | 359 ± 14.7 | 310-320 | <0.001 |
| Phosphorus (mg) | 1641 ± 24 | 1886 ± 124 | 700^2^ | 0.091 |  | 1155 ± 18 | 1339 ± 61 | 700 | 0.004 |
| Potassium (mg) | 3604 ± 52 | 4189 ± 272 | 4700^4^ | 0.035 |  | 2741 ± 39 | 3060 ± 130 | 4700^4^ | 0.019 |
| Sodium (mg) | 3719 ± 62 | 3241 ± 326 | 1200-1500^4^ | 0.150 |  | 2545 ± 42 | 2206 ± 141 | 1200-1500^4^ | 0.021 |
| Zinc (mg) | 15.1 ± 0.4 | 12.3 ± 2.2 | 11^2^ | 0.198 |  | 10.1 ± 0.3 | 9.5 ± 1.0 | 8^2^ | 0.587 |
| Vitamin A (RE)^5^ | 1314 ± 56 | 1772 ± 291 | 900 RAE^2,6^ | 0.168 |  | 1186 ± 53 | 1208 ± 175 | 700 RAE^2,6^ | 0.908 |
| Vitamin C (mg) | 130 ± 4.7 | 197 ± 24.8 | 90^2^ | 0.009 |  | 109 ± 3.6 | 125 ± 12.1 | 75^2^ | 0.203 |
| Thiamin (mg) | 2.1 ± 0.04 | 2.1 ± 0.2 | 1.2^2^ | 0.646 |  | 1.4 ± 0.03 | 1.7 ± 0.08 | 1.1^2^ | 0.003 |
| Riboflavin (mg) | 2.3 ± 0.04 | 2.6 ± 0.2 | 1.3^2^ | 0.161 |  | 1.7 ± 0.02 | 1.7 ± 0.09 | 1.1^2^ | 0.356 |
| Niacin (mg) | 49.0 ± 0.7 | 38.4 ± 3.8 | 16^2^ | 0.007 |  | 31.2 ± 0.5 | 34.2 ± 1.6 | 14^2^ | 0.070 |
| Pantothenate (mg) | 6.2 ± 0.1 | 6.2 ± 0.5 | 5^4^ | 0.982 |  | 4.2 ± 0.1 | 5.1 ± 0.2 | 5^4^ | 0.001 |
| Vitamin B_6_ (mg) | 2.3 ± 0.04 | 2.2 ± 0.2 | 1.3-1.7^2^ | 0.822 |  | 1.5 ± 0.02 | 1.7 ± 0.08 | 1.3-1.5^2^ | 0.008 |
| Folate (mcg DFE)^7^ | 556 ± 10 | 608 ± 53 | 400^2^ | 0.336 |  | 372 ± 7 | 460 ± 22 | 400^2^ | <0.001 |
| Vitamin B_12_ (mcg) | 5.0 ± 0.2 | 3.5 ± 0.8 | 2.4^2^ | 0.062 |  | 3.2 ± 0.1 | 2.9 ± 0.3 | 2.4^2^ | 0.308 |

1. Recommended Intake
2. Recommended Dietary Allowance
3. Acceptable Macronutrient Distribution Range
4. Adequate Intake
5. Retinol Equivalents
6. Retinol Activity Equivalents
7. Dietary Folate Equivalents
